# Menopause Predisposes Women to Increased Risk of Cardiovascular Disease

**DOI:** 10.3390/jcm12227058

**Published:** 2023-11-13

**Authors:** Magdalena Sylwia Kamińska, Daria Schneider-Matyka, Kamila Rachubińska, Mariusz Panczyk, Elżbieta Grochans, Anna Maria Cybulska

**Affiliations:** 1 Subdepartment of Long-Term Care and Palliative Medicine, Department of Social Medicine, Faculty of Health Sciences, Pomeranian Medical University in Szczecin, 48 Żołnierska St., 71-210 Szczecin, Poland; 2 Department of Nursing, Faculty of Health Sciences, Pomeranian Medical University in Szczecin, 48 Żołnierska St., 71-210 Szczecin, Poland; 3 Department of Education and Research in Health Sciences, Faculty of Health Sciences, Medical University of Warsaw, 14/16 Litewska St., 00-518 Warsaw, Poland

**Keywords:** cardiovascular disease, coronary heart disease, diagnostic medicine, medical risk factors, menopause, women’s health

## Abstract

(1) Background: Menopause is an important event in women’s lives, possibly contributing to the development of CVD, which is associated with changes in the cardiovascular risk profile, markers of metabolic health, and subclinical atherosclerosis. The aim of this study was to assess the association of menopause with CVD risk factors and subclinical markers of cardiometabolic disease. (2) Methods: The study involved 235 women from the general population at different stages of menopause. The methods used in this study were: diagnostic survey, anthropometric measurement (WC, height, BMI, WHtR), blood pressure measurement, biochemical analysis of venous blood (lipid profile, glucose, insulin, HbA1c), and CVD risk assessment (ASCVD Risk Calculator, POL-SCORE, SCORE-2). (3) Results: The vast majority of respondents had low cardiovascular risk, irrespective of the scale used for measuring the risk of CVD. The age at menopause was not an independent risk factor for CVD. In Model 1, the age at menopause and the time since menopause were found to be factors that increased CVD risk (OR = 1.186 and 1.267, respectively). In Models 2 and 3, the severity of menopausal symptoms was not a risk factor for CVD. Models 3 and 4 demonstrated that women with metabolic syndrome (MetS) were at a significantly higher risk of CVD. In model 5, the odds ratio of CVD with MetS as a standalone factor was 13.812. (4) Conclusions: Menopause predisposes women to an increased risk and MetS to a significantly higher risk of CVD.

## 1. Introduction

Cardiovascular disease (CVD) is currently the leading cause of death among women [1], accounting for 50% of cases, of which 20% are attributable to ischemic heart disease (IHD) and 13% to stroke [2]. 

Menopause is an important event in women’s lives, possibly contributing to the development of CVD, which is associated with changes in the cardiovascular risk profile, markers of metabolic health, and subclinical atherosclerosis [3]. As early as 1976, the Framingham Heart Study researchers reported a 2.6-fold higher incidence of cardiovascular events in postmenopausal women than in their premenopausal counterparts of the same age [4]. What is more, women develop CVD on average 7–10 years later than men, mainly due to the protective effect of estrogens on the atherosclerotic process [5]. The risk of CVD increases with the onset of menopause, and this is mainly seen among women with early menopause (EM) (i.e., when the age at menopause is <45 years) or premature ovarian insufficiency (POI), which is diagnosed if the age at menopause is <40 years) [6]. This is mainly due to specific biological factors or unique clinical conditions that pose additional cardiovascular risks. 

Menopause involves atherogenic changes in the lipid profile. Postmenopausal women have higher levels of total cholesterol, triglycerides, and low-density lipoprotein (LDL-C), as well as reduced levels of high-density lipoprotein (HDL-C) [7]. In addition, after menopause, the risk of developing metabolic syndrome (MetS) increases because of impaired glucose metabolism, weight gain, and central abdominal obesity [7,8]. Studies have shown several important features of menopause that affect cardiovascular risk [9,10]:

- Age at menopause: Women who last menstruated before the age of 45 have a higher overall risk of and mortality from ischemic heart disease. In addition, women experiencing early menopause (age of 40–45) and women with premature menopause (under 40) are at higher risk of heart disease.
- Type of menopause: The risk of CVD is higher for women whose menopause is the result of bilateral oophorectomy without estrogen therapy (especially women under 40 years of age).
- Stage of menopause: The highest blood pressure, cholesterol, and triglyceride values are recorded during either late perimenopause or early post-menopause.
- Vasomotor symptoms: The presence of vasomotor symptoms and other menopausal symptoms is associated with an increased risk of CVD and stroke.
- Sleep disorders: The combination of hormonal fluctuations, life stressors, and hot flashes contributes to sleep disorders, which in turn are associated with poorer cardiovascular health.
- Changes in estrogen levels have also a significant impact on the occurrence of CVD. This is because estrogens regulate various systemic factors, affecting, for example, serum lipid concentrations, the coagulation and fibrinolysis system, the antioxidant system, and the production of vasoactive molecules—nitric oxide and prostaglandins [11].
- Depression: Studies show that the development of depressive symptoms during the menopausal transition is associated with an increased risk of CVD [12].
- Other health changes associated with menopause: increased lipid levels, MetS, increased carotid atherosclerosis, weight gain, and ectopic fat distribution.

Other important factors increasing the risk of CVD in women are type 2 diabetes, hypertension, and obesity. Women with diabetes have a 1.81-fold higher risk of death from ischemic heart disease and a five-fold higher risk of heart failure than their non-diabetic counterparts [13]. Women with hypertension have higher population-adjusted cardiovascular mortality than men and are less likely to be treated according to blood pressure guidelines [14]. Postmenopausal women are especially prone to CVD—as estrogen levels decline, so does their protective vasodilating effect and the resulting blood pressure benefits [15]. Obesity is another traditional cardiovascular risk factor that is more common in women than in men [16]. The Framingham Heart Study revealed that obesity raises the relative risk of coronary artery disease (CAD) by 64% in women compared with 46% in men [14,17]. 

CVD is a serious problem among women entering menopause. The changing hormonal environment predisposes them to an increased risk of CVD due to the accumulation of risk factors such as visceral obesity, dyslipidemia, impaired glucose homeostasis, hypertension, and non-alcoholic fatty liver disease. Therefore, the aim of our study was to analyze the association of menopause with CVD risk factors and subclinical markers of cardiometabolic disease. In addition, this study assessed the prevalence of cardiovascular risk factors and the 10-year risk of CVD in peri- and postmenopausal women. 

## 2. Materials and Methods

### 2.1. Organization and Course of the Study

**Figure 1:**
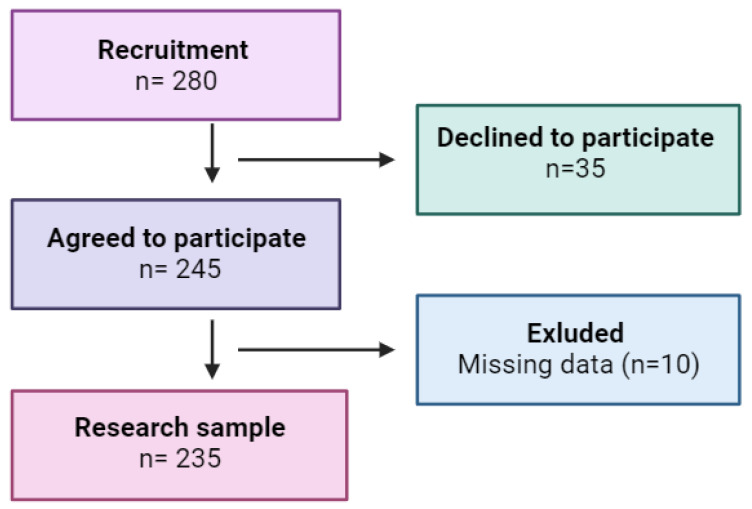
Study flow chart.

Our research was conducted in the West Pomeranian Voivodeship and recruited 280 women from the general population, of whom 235 were eventually involved (Figure 1). The criteria for inclusion in the study were female sex, age between 45 and 65 years, and informed written consent to take part in it. The criteria for exclusion from the study were current thyroid, neoplastic, or mental diseases or history of these conditions, and using menopausal hormone therapy (MHT) in a form other than pills (for example hormone patches or suppositories).

This was a cross-sectional study based on non-random convenience sampling. Participants were recruited by means of information posters left in public places and advertisements in local newspapers.

The project was approved by the Bioethics Committee of the Pomeranian Medical University in Szczecin (KB-0012/181/13) and performed in accordance with the Declaration of Helsinki.

This study is part of a larger project, whose aim is to evaluate the health of peri- and postmenopausal women from the West Pomeranian Voivodeship. 

### 2.2. Design of the Study

The study was carried out in four stages. The methods used in this study were diagnostic survey, anthropometric measurements, blood pressure measurement, and laboratory analysis. 

#### 2.2.1. Research Tools

Data collection was carried out using the authors’ questionnaire concerning basic sociodemographic variables (age, place of residence, employment status, education, marital status), stimulants (alcohol, tobacco), and health (menstruation, inflammation, mental diseases, cancer, menopause). Standardized research tools were also employed, namely the Beck Depression Inventory-I (BDI-I) and the Blatt–Kupperman Menopausal Index (BKMI):

- The BDI-I is a 21-item, multiple-choice self-report questionnaire to assess the severity of depressive symptoms. A four-point Likert scale (0–3) is used to measure each BDI-I item over the past two weeks. The higher the score, the more severe the depressive symptoms. A total score of 0–11 indicates no depressive symptoms, 12–19—mild depression, 20–25—moderate depression, and 26–63—severe depression. Cronbach’s alpha for the BDI-I total score is 0.89 [18].
- The BKMI is a tool to assess the severity of 11 climacteric symptoms: hot flashes, sweating, insomnia, nervousness, melancholy, dizziness, weakness, joint pain, headache, palpitations, and paresthesia. Each of them is scored from 0 to 3, indicating no, mild, moderate, and severe symptoms, respectively. The symptoms are weighted: hot flashes (×4), paresthesia (×2), insomnia (×2), nervousness (×2), and all other symptoms (×1); therefore, the highest potential score is 51. The results are interpreted as follows: 0–16 points—no symptoms, 17–25 points—mild symptoms, 26–30 points—moderate symptoms, above 30 points—severe symptoms of menopause [19].

#### 2.2.2. Anthropometric Measurements

The next step was anthropometric measurements (waist circumference, weight, height):

- Waist circumference (WC) was measured to the nearest 0.01 m using a flexible tape measure (SECA 711). Waist circumference was measured as the horizontal distance around the abdomen at the level of the navel. Abdominal obesity was defined as WC ≥ 80 cm (for European women) [20];
- A validated medical scale with an integrated SECA 711 height meter was used to measure body weight and height in accordance with a standardized procedure with an accuracy of 0.1 kg and 0.1 cm, respectively. Participants stood with their backs straight, heels together, barefoot, in light clothing. Based on the results, the body mass index (BMI) was calculated: BMI = weight [kg]/height [m^2^]. Based on the BMI values (kg/m^2^), the following categories were established as recommended by the Centers for Disease Control and Prevention (CDC): underweight (BMI < 18.5), normal weight (BMI = 18.5–24.9), overweight (BMI = 25.0–29.9), obesity (BMI ≥ 30) [21].
- Waist-to-height ratio (WHtR) was determined according to the formula: WHtR = waist circumference [cm]/height [cm]) [20].

#### 2.2.3. Blood Pressure Measurements

The Korotkoff sound technique was used to measure blood pressure (BP). We took care to ensure that the patient was in the correct position, had a period of quiet rest, and used an appropriately sized cuff. External factors affecting blood pressure such as smoking and consuming caffeinated products before measuring blood pressure were minimized [22]. We followed the recommendations of the American Heart Association [23,24]. 

#### 2.2.4. Laboratory Analysis

Blood was taken from each of the volunteers after an overnight fast between 7 a.m. and 9.30 a.m. after a 10 min sitting rest. It was drawn from the ulnar vein using a vacutainer system to assess baseline biochemical parameters, such as insulin, glucose, glycated hemoglobin (HbA1c), total cholesterol (TC), HDL-C, LDL-C, and triglycerides (TG). 

Blood was collected by qualified nurses in accordance with the rules and procedures for the collection, storage, and transport of biological material. Biochemical parameters were determined in a certified laboratory of the Pomeranian Medical University in Szczecin using standard commercial methods. 

### 2.3. Cardiovascular Risk Assessment

The study used three scales to assess cardiovascular risk:

- POL-Systematic Coronary Risk Evaluation (POL-SCORE) 2015—a tool to assess ten-year risk of a fatal cardiovascular event with regard to sex, age, systolic blood pressure, total cholesterol, and smoking—the version for the Polish population. The risk of cardiovascular death within ten years according to the POL-SCORE was as follows: low < 1%, moderate 1–4%, high 5–9%, and very high ≥ 10% [25];
- SCORE-2—an updated predictive model to estimate the ten-year risk of death from CVD and non-fatal CVD in 40–69-year-old Europeans without prior CVD or diabetes. This version was developed in 2021 for four risk groups: low, moderate, high, and very high. Poland is recognized as one of the countries at high risk of CVD [26,27];
- Atherosclerotic Cardiovascular Disease (ASCVD) Risk Calculator 2013—a tool recommended by the Framingham Heart Study to calculate 10-year ASCVD risk). The calculator was developed by the American Heart Association and the American College of Cardiology [28,29].

### 2.4. Classification of Respondents

The study recruited 280 women between the ages of 45 and 65, representing the general population of the West Pomeranian Voivodeship in northwestern Poland. Ultimately, 235 respondents who met all the inclusion criteria were included in the study (completion rate: 84%).

The size of the study sample was determined on the basis of statistical data on the population of women aged 45–64 in the West Pomeranian Voivodeship in 2020 [30]. The confidence level was set at 95%, the maximum error at 7%, and the estimated fraction size at 0.5. 

The women were divided into groups depending on their:

(a) Menopausal status [31]:
  - Perimenopause—the time immediately before menopause, when endocrine, biological, and clinical symptoms of approaching menopause begin;
  - Postmenopause—last menstrual period (at least 12 months before the examination);
(b) Hypertension—diagnosis based on the 2020 International Society of Hypertension Global Hypertension Practice guidelines and the 2019 Polish Society of Hypertension guidelines (systolic blood pressure (SBP) ≥ 140 mmHg or diastolic blood pressure (DBP) ≥ 90 mmHg or taking antihypertensive drugs) [22,23];
(c) Obesity—diagnosis based on the CDC recommendations (underweight: BMI < 18.5, normal weight: BMI = 18.5–24.9, overweight: BMI = 25.0–29.9, obesity: BMI ≥ 30, general obesity: ≥30 kg/m^2^, and abdominal obesity: WC > 80 cm) [20];
(d) MetS—based on the latest criteria proposed by the International Diabetes Federation (IDF) and the modified National Cholesterol Education Program Adult Treatment Panel III [32], a woman is diagnosed with MetS if she has three out of five risk factors, which include:
  - WC ≥ 80 cm,
  - TG > 150 mg/dL (1.7 mmol/L) or treatment of this lipid abnormality,
  - HDL-C < 50 mg/dL (1.3 mmol/L) or treatment of this lipid abnormality,
  - Elevated BP: SBP ≥ 130 or DBP ≥ 85 mmHg or treatment of previously diagnosed hypertension,
  - Fasting plasma glucose (FPG) ≥ 100 mg/dL (5.6 mmol/L) or previously diagnosed type 2 diabetes. If it is above 5.6 mmol/L or 100 mg/dL, an oral glucose tolerance test (OGTT) is strongly recommended, but this is not necessary to determine the presence of the syndrome;
(e) Pre-metabolic syndrome (pre-MetS)—defined as having at least two components of MetS, but not meeting the above criteria for the diagnosis of MetS [33,34];
(f) Dyslipidemia—based on the National Cholesterol Education Program (NCEP) guidelines, diagnosed if [35]:
  - total cholesterol (TC) ≥ 240 mg/dL;
  - low-density lipoprotein cholesterol (LDL-C) ≥ 160 mg/dL;
  - triglyceride (TG) level ≥ 88 mg/dL;
  - high-density lipoprotein cholesterol (HDL-C) ≤ 40 mg/dL or taking lipid-regulating drugs.

### 2.5. Statistical Analysis

Quantitative, nominal, and ordinal variables were described with descriptive statistical methods. The following measures were determined for quantitative variables: central tendency (mean, M) and dispersion (standard deviation, SD). For nominal and ordinal variables, the following measures were determined: number (n) and frequency (%). Cross-tables and the Pearson’s chi-squared test with odds ratio were used to assess frequency difference for variants of categorical variables. To assess differences in selected quantitative variables between perimenopausal and postmenopausal women, Student’s *t*-test with calculation of the mean difference was used.

All calculations were performed with Statistica v. 13.3 (TIBCO Software, Palo Alto, CA, USA). Statistical significance was set at *p* < 0.05.

## 3. Results

### 3.1. Menopausal Status and Cardiovascular Risk 

The study assessed CVD risk among women. Diabetes was diagnosed in 6.36% of them, hypertension in 54.34%, hyperlipidemia in 78.04%, MetS in 12.4%, and polycystic ovary syndrome (PCOS) in one woman. Some 19.08% smoked cigarettes, and 16.8% consumed alcohol. General obesity was observed in 31.21% of postmenopausal women, whose mean BMI was 28.23 (SD = 5.33). The mean glucose levels were 93.27 mg/dL, cholesterol—210.51 mg/dL, HDL-C—66.98 mg/dL, and LDL-C—123.11 mg/dL (Table 1 and Table 2).

Table 1 and Table 2 show the anthropometric and clinical characteristics of the respondents, taking into account their menopausal status. Postmenopausal women achieved significantly higher POL-SCORE 2015 (*p* < 0.001) and BKMI (*p* = 0.013) results. It is noteworthy that the vast majority of the women had no climacteric symptoms. Severe climacteric symptoms were observed in 3.23% of perimenopausal and 4.05% of postmenopausal women (Table 2). 

In this study, CVD risk was assessed using the ASCVD Risk Calculator, POL-SCORE 2015, and SCORE-2. It was observed that the vast majority of the respondents had low CVD risk, irrespective of the scale that was used to assess it (Table 3).

### 3.2. Impact of Menopause on CVD Risk According to POL-SCORE 2015

#### 3.2.1. POL-SCORE 2015

This study assessed the impact of menopause on CVD risk with regard to the POL-SCORE 2015 scores. 

In Model 0, in which the influence of other factors (e.g., abdominal obesity, general obesity) was not taken into account, the odds of CVD risk according to POL-SCORE 2015 were significantly higher in the menopausal group than in the non-menopausal one. In Model 1, the odds of CVD were not related to obesity. In Model 2, HbA1c was a factor increasing the risk of CVD (OR = 1.026) among women. Model 3 and Model 4 showed that women with MetS had significantly higher likelihood of CVD (Table 4).

#### 3.2.2. SCORE-2

This study evaluated the impact of menopause on CVD risk, taking into account SCORE-2 results. 

Model 0 demonstrated that menopause was not an independent risk factor for CVD. In Model 1, obesity was not found to be a factor increasing the risk of CVD in women. In Model 2, TG levels were a CVD risk factor. Model 3 showed that women with MetS were at a significantly higher risk of CVD. In Model 4, women with MetS were found to have a significantly higher CVD risk (Table 5).

### 3.3. Impact of Menopause on CVD Risk According to the ASCVD Risk Calculator Score 

The study evaluated the impact of menopause on cardiovascular risk with regard to the ASCVD Risk Calculator results. For Model 1, it was observed that general obesity was a factor increasing CVD risk in women. 

Model 2 confirmed that HbA1c was a risk factor for CVD. In Model 3, women with MetS had a significantly higher CVD risk. In Model 4, women with MetS and general obesity were found to be at a significantly higher risk of CVD (Table 6). 

### 3.4. Impact of Menopause-Related Variables on the Occurrence of CVD 

#### 3.4.1. POL-SCORE 2015

This study evaluated the impact of menopause-related variables on cardiovascular risk, taking into account the POL-SCORE 2015 results. 

Based on the collected data (Model 0), it was shown that the age at menopause was not an independent risk factor for CVD. In Model 1, the age at menopause and the time since menopause were CVD risk factors (OR = 1.382 and 1.471, respectively). Model 2 revealed that the severity of menopausal symptoms was not a CVD risk factor. Model 3 and Model 4 proved that women with MetS were at a significantly higher risk of CVD (Table 7).

#### 3.4.2. ASCVD Risk Calculator

This study assessed the impact of menopause-related variables on cardiovascular risk, taking into account the ASCVD Risk Calculator results. 

In Model 0, the age at menopause was not an independent risk factor for CVD. In Model 1, the age at menopause and the time since menopause were factors increasing CVD risk (OR = 1.186 and 1.267, respectively). Models 2 and 3 showed that the severity of menopausal symptoms was not a risk factor for CVD. Models 3 and 4 demonstrated that women with MetS were at a significantly higher risk of CVD. In Model 5, the odds ratio of CVD with MetS as a standalone factor was OR = 13.812 (Table 8).

## 4. Discussion

The purpose of this study was to assess the association of menopause with CVD risk factors and subclinical markers of cardiometabolic disease. We are the first to analyze the correlation between menopausal status and CVD risk factors, especially between cardiovascular risk factors and the 10-year risk of CVD in Polish peri- and postmenopausal women.

### 4.1. Effect of Menopause on CVD Risk

Middle-aged women are at higher risk of CVD than men. Traditional CVD risk factors affect women more strongly, contributing to this difference. Epidemiological studies have shown that menopausal transition is associated with a higher prevalence of CVD risk factors [36]. However, the impact of menopause on CVD risk in women has not been fully elucidated [36]. 

Menopause affects many metabolic biomarkers more than the process of aging [37]. Postmenopausal women experience changes in the metabolism of lipoproteins, fatty acids, and amino acids. These changes are independent of age, and potentially underlie the link between menopause and cardiometabolic diseases. 

As stated by Gurka et al. [38], the severity of MetS and five risk factors for heart disease may increase before menopause faster than after menopause. Women may exhibit vasomotor or other menopausal symptoms before menstruation ceases [39]. 

For many years, researchers have been trying to understand the impact of selected determinants of increased CVD risk in postmenopausal women [40]. It has been observed that in addition to the accumulation of CVD risk factors during menopause (e.g., increased fasting blood glucose, higher blood pressure, and increased blood lipid levels), the incidence of type 2 diabetes, which is a major CVD risk factor, also increases [9]. In addition, menopausal symptoms, such as hot flashes, night sweats, sleep disturbances, depression, and anxiety are associated with an unfavorable cardiometabolic profile, and thus with an increased risk of CVD [41]. 

Hyperandrogenism in peri- and postmenopausal women has been found to be associated with an unfavorable cardiovascular risk profile [42,43,44].

Environmental and lifestyle factors (diet, alcohol consumption, physical activity, BMI, smoking, socio-demographic, psychological, and socio-cognitive factors) can affect the occurrence of CVD in women [45]. These variables may trigger mechanisms underlying menopause-related CVD.

In a study by Chen et al., the age at menopause (whether early or late menopause) was found to be less important as a CVD risk factor than menopause itself [46]. It has been proven that premenopausal women with vasomotor or nonvasomotor menopausal symptoms exhibit a significantly higher prevalence of poor cardiovascular health (CVH) compared with those without any menopausal symptoms [47]. The SWAN and MsHeart/MsBrain studies have demonstrated that frequent and/or persistent vasomotor symptoms (VMS) correlate with unfavorable CVD risk factor profiles, reduced peripheral vascular and cerebrovascular health, and increased risk of clinical CVD events [48].

Our study shows that in Model 0, which did not account for the influence of other factors, the likelihood of CVD risk according to POL-SCORE was significantly higher in the menopausal group than in the non-menopausal one. Considering the POL-SCORE-2 results, Model 0 showed that menopause was not an independent risk factor for CVD. After adjusting the odds for the age of the subjects and the presence of other variables predisposing to CVD (e.g., obesity, hypertension, hyperlipidemia, type 2 diabetes), there were no statistically significant differences in the severity of CVD risk between pre- and postmenopausal women.

The 1976 Framingham study provided some of the first evidence of changes in the severity of CVD risk in postmenopausal women. Indeed, more cardiovascular events were observed in postmenopausal women compared with premenopausal women of the same age [4].

Our study assessing the impact of menopause on CVD risk with regard to the POL-SCORE 2015 results indicated that in Model 1, the odds of CVD were not related to obesity, in Model 2, HbA1c was a factor increasing the risk of CVD in women, and Model 3 and Model 4 showed that women with MetS had a significantly higher risk of CVD. Taking into account POL-SCORE-2 results, in Model 2, TG levels were a CVD risk factor, Model 3 showed that women with MetS were at a significantly higher risk of CVD, and in Model 4, women with MetS were found to have a significantly higher CVD risk. A study by Ama Moor et al. [49] demonstrated that a large number of postmenopausal women are at risk of developing CVD. What is more, marital status, the levels of FPG and LDL-C, as well as DBP values can significantly affect CVD risk. In their study of diabetic women, Zhou et al. [50] observed that the risk of CVD was higher for postmenopausal women than for their premenopausal counterparts, and that factors significantly contributing to this phenomenon were LDL-C and FPG levels. Also, Shen et al. [51] indicate that postmenopausal status shows a significant relationship with worse glycemic levels. 

At the clinical level, glucose concentrations may remain unchanged or may become unstable, possibly leading to impaired glucose tolerance in women entering menopause [52]. A meta-analysis by Anagnostis et al. [36] showed that postmenopausal women with a history of early menopause or premature ovarian insufficiency have a higher risk of type 2 diabetes compared with women who experienced menopause after the age of 45. As confirmed by Muka et al. in their meta-analysis [9], climacteric symptoms are associated with an increased risk of CVD. On the other hand, Huang et al. [53] assert that there is a relationship between menopausal symptoms and the risk of coronary heart disease. These authors observed that the incidence of coronary heart disease was higher in the group with climacteric symptoms than in the control group. After adjusting for possible confounding factors in different models, the group with climacteric symptoms had a 1.338–1.441 times higher risk of coronary heart disease than the control cohort. 

### 4.2. Effect of the Time since Menopause on CVD Risk 

The risk of CVD increases with the passage of time since menopause [50]. There is probably a link between the time since menopause and some traditional CVD risk factors, such as MetS and obesity [54]. Cho et al. [55], on the other hand, noted that women who were 10–14 years after menopause were more likely to have higher TG levels.

Our study evaluated the impact of menopause-related variables on cardiovascular risk, taking into account the POL-SCORE results and the ASCVD Risk Calculator results. In both cases, based on the collected data (Model 0), the age at menopause was not an independent risk factor for CVD. Women approaching menopause are at increased risk of CVD, which is attributed to several factors, such as visceral obesity, atherogenic dyslipidemia, disruptions in glucose regulation, non-alcoholic fatty liver disease (NAFLD), and hypertension. Nevertheless, it remains controversial whether menopause itself directly contributes to the increased likelihood of CVD events [36]. It is worth noting that a clear association between menopause itself and an elevated risk of CVD events has only been established in the case of early menopause (occurring before the age of 45) [56].

We have not found any information indicating a connection between experiencing natural menopause at a later age and the risk of CVD. A review of the literature suggests that early natural menopause is a factor potentially increasing the likelihood of CVD. For example, Zhu et al. [57] reported that in comparison to women who underwent menopause at the age of 50–51 years, women who experienced premature or early menopause were at a significantly elevated risk of a non-fatal CVD event before reaching 60 years of age, although it was no longer observed after the age of 70 years. Hu et al. [58] noted a substantial association between a younger age at menopause and a higher risk of coronary heart disease (CHD) among women who had experienced natural menopause and had never used MHT. This increased risk was particularly noticeable among current smokers. The increased risk of CHD among smokers, especially those experiencing natural menopause at a younger age, may be associated with other smoking-related confounders. 

Some researchers speculate that the age of menopause contributes to CVD risk, and this relationship is bidirectional. The Framingham Heart Study revealed that higher total cholesterol, SBP, and DBP, as well as other CVD risk factors are associated with earlier menopause, irrespective of smoking [59]. Moreover, it was observed that the first CVD event before the age of 35 was associated with a doubling of the risk of early menopause, while the occurrence of a CVD event after the age of 35 was associated with menopause at the age of 51 [60]. It is therefore likely that poorer cardiovascular health before menopause may influence the onset of natural menopause. The age at which women reach natural menopause is considered a predictor of reproductive, somatic, and cardiometabolic aging, as well as women’s overall health. Late age of natural menopause has been linked to both positive (e.g., reduced overall mortality) and negative (e.g., higher risk of breast and ovarian cancer) health consequences [61]. 

Also, Tao et al. [62] confirm that premature ovarian insufficiency increases the risk of death from cardiac causes. A meta-analysis by Muka et al. [9] has provided evidence that early menopause is associated with a 1.5-fold increase in the risk of ischemic heart disease compared with the normal age at menopause (>45 years), while a meta-analysis by Armeni et al. [63] has shown that the relationship between vasomotor symptoms (VMS) and CVD events varies by age in peri- and postmenopausal women. Vasomotor symptoms increase the incidence of CVD events only in women younger than 60. 

## 5. Conclusions

- Menopause predisposes women to an increased risk of CVD due to visceral obesity, dyslipidemia, impaired glucose homeostasis, and hypertension. Also, women with MetS have a significantly higher risk of CVD.
- Menopause is associated with an increased risk of CVD. Despite many studies, it is difficult to clarify the dilemma regarding the independent and causal role of menopause in the development of CVD, taking into account the interaction of climacteric symptoms with traditional cardiovascular risk factors. It is, therefore, important to conduct research that will explain the complex mechanical pathways that may increase cardiometabolic risk after menopause.
- Further research evaluating the impact of selected variables on the occurrence of cardiovascular risk among peri- and postmenopausal women is recommended.

## 6. Limitation

Our study has some limitations. These include:

- The small size of the study sample—a larger number of participants would strengthen the study;
- The method of recruitment (posters and advertisements), and the fact that recruitment was limited to one voivodeship, which prevented us from reaching a wider group of potential participants;
- Lack of detailed history of unfavorable pregnancy outcomes and their complications, failure to assess family history of CVD, and lack of information about the first menstruation;
- Amenorrhea for at least 12 months was diagnosed on the basis of a gynecological history, but not confirmed by the measurement of FSH levels;
- We only included women who were not taking MHT, so we could not check whether MHT had any protective effect on the cardiovascular system.

## Figures and Tables

**Table 1:** Baseline characteristics of the participants (N = 235).

Variables	Perimenopausal (*n* = 62)		Postmenopausal (*n* = 173)		t_df=233_	*p*-Value *
	M	SD	M	SD		
Age (years)	48.73	2.96	56.44	4.13	−13.502	<0.001
Age at menopause (years)	-	-	48.76	4.45	-	-
Time since menopause (years)	-	-	7.68	5.09	-	-
Body mass (kg)	75.92	17.74	74.39	13.75	0.694	0.488
BMI (kg/m^2^)	28.34	5.99	28.23	5.33	0.137	0.891
WC (cm)	88.19	13.11	89.58	12.15	−0.761	0.447
WHtR	0.54	0.08	0.55	0.08	−1.029	0.305
HbA1c	5.48	1.10	5.58	0.95	−0.712	0.477
Fasting serum glucose (mg/dL)	90.93	38.16	93.27	35.85	−0.433	0.665
Insulin (µIU/L)	9.45	6.67	10.05	6.08	−0.651	0.516
Systolic BP (mmHg)	117.85	17.01	123.00	19.57	−1.836	0.068
Diastolic BP (mmHg)	77.37	10.02	77.97	10.45	−0.392	0.695
Total cholesterol (mg/dL)	214.31	30.36	210.51	37.21	0.723	0.471
LDL-C (mg/dL)	123.59	32.64	123.11	32.19	0.100	0.921
HDL-C (mg/dL)	70.96	17.11	66.98	18.32	1.494	0.137
TG (mg/dL)	90.57	37.06	102.53	46.90	−1.815	0.071
TG/HDL ratio	1.43	0.92	1.73	1.17	−1.800	0.073
TC/HDL ratio	3.19	0.85	3.32	0.90	−1.031	0.303
LDL/HDL ratio	1.88	0.74	1.98	0.75	−0.918	0.359
HOMA-IR	2.52	4.26	2.47	2.26	0.110	0.913
POL-SCORE 2015 (score)	0.29	0.49	1.06	1.42	−4.172	<0.001
BDI (score)	7.84	5.72	7.40	7.35	0.421	0.674
BKMI (score)	18.27	5.48	20.31	5.48	−2.504	0.013

BMI—body mass index, WC—waist circumference, WHtR—waist-circumference-to-height ratio, HbA1c—glycated hemoglobin, BP—blood pressure, LDL-C—low-density lipoprotein, HDL-C—high-density lipoprotein, TG—triglycerides, TC—total cholesterol, HOMA-IR—Homeostatic Model Assessment for Insulin Resistance, BDI—Beck Depression Inventory, BKMI—Blatt–Kupperman Menopausal Index, * Student’s *t*-test, M—mean, SD—standard deviation, N—number of all participants, *n*—number of participants in a subgroup, *p*—significance level.

**Table 2:** Distribution of CVD risk factors among different groups (%).

Variables	All (N = 235)		Perimenopausal (*n* = 62)		Postmenopausal (*n* = 173)		χ^2^	*p*-Value ^
	n	%	n	%	n	%		
Hypertension	115	48.94	21	33.87	94	54.34	7.649	0.006
Hyperlipidemia	183	77.87	48	77.42	135	78.03	0.010	0.920
Current smoking	46	19.57	13	20.97	33	19.08	0.104	0.747
Diabetes mellitus	13	5.53	2	3.23	11	6.36	0.857	0.355
General obesity	74	31.49	20	32.26	54	31.21	0.045	0.832
Abdominal obesity	120	51.06	29	46.77	91	52.60	0.620	0.431
BDI—no depression	178	75.74	48	77.42	130	75.14	1.842	0.606
BDI—mild depression	45	19.15	12	19.35	33	19.08		
BDI—moderate depression	7	2.98	2	3.23	5	2.89		
BDI—severe depression	5	2.13	0	0.00	5	2.89		
BKMI—no climacteric symptoms	146	61.13	47	75.81	99	57.23	8.086	0.044
BKMI—mild symptoms	50	21.28	6	9.68	44	25.43		
BKMI—moderate symptoms	30	17.57	7	11.29	23	13.29		
BKMI—severe symptoms	9	3.83	2	3.23	7	4.05		
MetS—no MetS	41	17.45	15	24.19	26	15.03	4.601	0.100
MetS—pre-MetS	170	72.34	44	70.97	126	72.83		
MetS—MetS	24	10.22	3	4.84	21	12.14		

^ Pearson’s chi-squared test, BDI—Beck Depression Inventory, BKMI—Blatt–Kupperman Menopausal Index, MetS—metabolic syndrome, *n*—number of participants in a subgroup, %—percent of participants, *p*—significance level.

**Table 3:** CVD risk assessment.

Variables		All		Perimenopausal (*n* = 62)		Postmenopausal (*n* = 173)	
		*n*	%	*n*	%	*n*	%
ASCVD Risk Calculator	Low risk	204	86.8	60	96.8	144	83.2
	Moderate risk	22	9.3	1	1.7	21	12.1
	High risk	9	3.8	1	1.7	8	4.6
POL-SCORE 2015	Low risk	196	83.4	61	98.4	135	78.0
	Moderate risk	31	13.2	1	1.6	30	17.3
	High risk	8	3.4	0	0	8	4.6
SCORE-2 *	Low risk	187	84.23	58	96.67	129	79.63
	Moderate risk	29	13.06	2	3.33	27	16.67
	High risk	6	2.7	0	0	6	3.7

* exclusion of women with a diagnosis of type 2 diabetes, *n*—number of participants in a subgroup, %—percent of participants.

**Table 4:** Impact of CVD risk factors not included in POL-SCORE 2015 on the development of CVD.

		POL-SCORE 2015				
Model	Variables	OR	−95%CI	+95%CI	Wald’s Statistics	*p*-Value
Model 0	Intercept	0.016	0.002	0.118	16.627	<0.001
	Group (pre vs. post)	17.170	2.304	127.958	7.698	0.006
Model 1	Intercept	0.113	0.057	0.224	38.711	<0.001
	General obesity	2.115	0.507	8.814	1.058	0.304
	Abdominal obesity	1.858	0.908	3.801	2.880	0.090
Model 2	Intercept	0.005	0.000	0.145	9.609	0.002
	WC [cm]	1.015	0.987	1.044	1.091	0.296
	HbA1c [%]	1.394	1.026	1.895	4.510	0.034
	LDL-C [mg/dL]	1.000	0.989	1.012	0.001	0.972
	TG [mg/dL]	1.004	0.996	1.011	0.988	0.320
Model 3	Intercept	0.127	0.043	0.375	13.950	<0.001
	Diabetes	2.034	0.476	8.694	0.918	0.338
	MetS (no MetS vs. pre-MetS)	1.389	0.450	4.286	0.326	0.568
	MetS (no MetS vs. MetS)	8.765	2.074	37.040	8.714	0.003
	BDI score	0.975	0.922	1.031	0.807	0.369
Model 4	Intercept	0.103	0.033	0.328	14.846	<0.001
	General obesity	3.137	0.729	13.491	2.359	0.125
	Abdominal obesity	1.462	0.611	3.495	0.728	0.393
	Diabetes	2.083	0.484	8.962	0.972	0.324
	MetS (no MetS vs. pre-MetS)	1.011	0.280	3.653	0.000	0.987
	MetS (no MetS vs. MetS)	6.586	1.290	33.635	5.133	0.023
	BDI score	0.975	0.921	1.033	0.725	0.395

CI—confidence interval, WC—waist circumference, HbA1c—glycated hemoglobin, LDL-C—low-density lipoprotein cholesterol, TG—triglycerides, MetS—metabolic syndrome, BDI—Beck Depression Inventory, *p*—significance level. Model 0—model adjusted for the variable: menopausal status. Model 1—model adjusted for the variables: general and abdominal obesity. Model 2—model adjusted for the variables: WC, HBA1C, LDL-C, TG. Model 3—model adjusted for the variables: diabetes, MetS, BDI score. Model 4—model adjusted for the variables: general and abdominal obesity, diabetes, MetS, BDI score.

**Table 5:** Impact of CVD risk factors not included in SCORE-2 on the development of CVD.

Model	Variables	SCORE-2				
		Beta (β)	−95% CI	+95% CI	t	*p*
Model 0	Intercept				78.169	<0.001
	Group (pre vs. post)	0.067	−0.062	0.195	1.020	0.309
Model 1	Intercept				87.409	<0.001
	General obesity	0.085	−0.212	0.382	0.564	0.573
	Abdominal obesity	0.073	−0.055	0.201	1.117	0.265
Model 2	Intercept				8.623	<0.001
	WC [cm]	0.004	−0.101	0.109	0.076	0.940
	HbA1C [%]	0.073	−0.036	0.182	1.315	0.190
	TG [mg/dL]	0.563	0.454	0.672	10.166	<0.001
Model 3	Intercept				55.744	<0.001
	BDI (score)	0.004	−0.118	0.126	0.071	0.944
	MetS (no MetS vs. pre-MetS)	−0.230	−0.374	−0.087	−3.163	0.002
	MetS (no MetS vs. MetS)	0.412	0.268	0.556	5.634	<0.001
Model 4	Intercept				55.800	<0.001
	General obesity	0.136	−0.144	0.416	0.959	0.339
	Abdominal obesity	−0.047	−0.184	0.091	−0.671	0.503
	BDI (score)	−0.010	−0.131	0.112	−0.161	0.873
	MetS (no MetS vs. pre-MetS)	−0.205	−0.351	−0.060	−2.777	0.006
	MetS (no MetS vs. MetS)	0.445	0.295	0.595	5.852	<0.001

β—standardized regression coefficient, CI—confidence interval, WC—waist circumference, HbA1c—glycated hemoglobin, TG—triglycerides, BDI—Beck Depression Inventory, MetS—metabolic syndrome. Model 0—model adjusted for the variable: menopausal status. Model 1—model adjusted for the variables: general and abdominal obesity. Model 2—model adjusted for the variables: WC, HBA1C, LDL-C, TG. Model 3—model adjusted for the variables: diabetes, MetS, BDI score. Model 4—model adjusted for the variables: general and abdominal obesity, diabetes, MetS, BDI score.

**Table 6:** Impact of CVD risk factors not included in the ASCVD Risk Calculator on the development of CVD.

Model	Variables	ASCVD Risk Calculator				
		OR	−95%CI	+95%CI	Wald’s Statistics	*p*-Value
Model 1	Intercept	0.164	0.079	0.342	23.202	<0.001
	General obesity	0.000	0.000	0.000	1213.184	<0.001
	Abdominal obesity	1.128	0.502	2.535	0.086	0.770
Model 2	Intercept	0.001	0.000	0.041	11.431	0.001
	WC [cm]	1.000	0.964	1.038	0.000	0.987
	HbA1c [%]	2.748	1.626	4.643	14.258	<0.001
	TG [mg/dL]	1.001	0.992	1.011	0.085	0.771
Model 3	Intercept	0.077	0.017	0.343	11.309	0.001
	Mets (no MetS vs. pre-Mets)	1.484	0.316	6.972	0.250	0.617
	Mets (no MetS vs. Mets)	19.138	3.522	103.979	11.684	0.001
	BDI (score)	1.012	0.956	1.072	0.171	0.679
Model 4	Intercept	0.066	0.014	0.312	11.763	0.001
	General obesity	0.000	0.000	0.000	822.633	<0.001
	Abdominal obesity	0.562	0.205	1.540	1.254	0.263
	Mets (no MetS vs. pre-Mets)	1.794	0.342	9.418	0.478	0.489
	Mets (no MetS vs. Mets)	25.623	3.885	169.002	11.357	0.001
	BDI (score)	1.022	0.964	1.082	0.520	0.471

OR—odds ratio, CI—confidence interval, WC—waist circumference, HbA1c—glycated hemoglobin, TG—triglycerides, MetS—metabolic syndrome, BDI—Beck Depression Inventory. Model 0—could not be created because there was no increased risk of CVD among perimenopausal women. Model 1—model adjusted for the variables: general and abdominal obesity. Model 2—model adjusted for the variables: WC, HBA1C, TG. Model 3—model adjusted for the variables: diabetes, MetS, BDI score. Model 4—model adjusted for the variables: general and abdominal obesity, MetS, BDI score.

**Table 7:** Impact of menopause-related variables on CVD risk according to the POL-SCORE 2015 results.

Model	Variables	POL-SCORE					
		b	OR	−95%CI	+95%CI	Wald’s Statistics	*p*-Value
Model 0	Intercept	−2.556	0.078	0.001	4.429	1.535	0.215
	Age at menopause	0.026	1.027	0.946	1.115	0.395	0.529
Model 1	Intercept	−20.530	0.000	0.000	0.000	25.236	<0.001
	Age at menopause	0.324	1.382	1.200	1.592	20.145	<0.001
	Time since menopause (years)	0.386	1.471	1.283	1.686	30.624	<0.001
Model 2	Intercept	−20.015	0.000	0.000	0.000	23.144	<0.001
	Age at menopause	0.323	1.381	1.199	1.591	19.942	<0.001
	Time since menopause (years)	0.390	1.477	1.287	1.696	30.846	<0.001
	BKMI (score)	−0.026	0.975	0.897	1.059	0.365	0.546
Model 3	Intercept	−20.818	0.000	0.000	0.000	23.025	<0.001
	MetS (no MetS vs. pre-MetS)	0.237	1.267	0.294	5.467	0.101	0.751
	MetS (no MetS vs. MetS)	2.364	10.631	1.840	61.438	6.975	0.008
	Age at menopause	0.332	1.394	1.202	1.618	19.206	<0.001
	Time since menopause (years)	0.371	1.449	1.260	1.666	27.121	<0.001
	BKMI (score)	−0.028	0.973	0.891	1.062	0.388	0.534
Model 4	Intercept	−2.225	0.108	0.039	0.303	17.865	<0.001
	MetS (no MetS vs. pre-MetS)	0.318	1.375	0.447	4.234	0.308	0.579
	MetS (no MetS vs. MetS)	2.392	10.932	2.958	40.405	12.858	<0.001

OR—odds ratio, CI—confidence interval, b—nonstandardized regression coefficient, BKMI—Blatt–Kupperman Menopausal Index, MetS—metabolic syndrome. Model 0—model adjusted for the variable: age at menopause. Model 1—model adjusted for the variables: age at menopause, time since menopause [years]. Model 2—model adjusted for the variables: age at menopause, time since menopause [years], BKMI score. Model 3—model adjusted for the variables: MetS, age at menopause, time since menopause [years], BKMI score. Model 4—model adjusted for the variable: MetS.

**Table 8:** Impact of menopause-related variables on CVD risk according to the ASCVD Risk Calculator results.

Model	Variables	b	OR	−95%CI	+95%CI	Wald’s Statistics	*p*-Value
Model 0	Intercept					0.289	0.591
	Age at menopause	−0.008	0.992	0.907	1.085	0.033	0.856
Model 1	Intercept					11.476	0.001
	Age at menopause	0.170	1.186	1.047	1.343	7.156	0.007
	Time since menopause (years)	0.237	1.267	1.129	1.422	16.145	<0.001
Model 2	Intercept					11.796	0.001
	Age at menopause	0.173	1.189	1.050	1.347	7.439	0.006
	Time since menopause (years)	0.233	1.263	1.125	1.417	15.738	<0.001
	BKMI (score)	0.027	1.027	0.949	1.112	0.443	0.506
Model 3	Intercept					11.478	0.001
	MetS (no MetS vs. MetS)	2.593	13.371	4.260	41.964	19.747	<0.001
	Age at menopause	0.188	1.207	1.052	1.385	7.206	0.007
	Time since menopause (years)	0.214	1.238	1.097	1.398	11.923	0.001
	BKMI (score)	0.029	1.029	0.945	1.121	0.438	0.508
Model 4	Intercept					11.969	0.001
	Abdominal obesity (no vs. yes)	−0.832	0.435	0.153	1.236	2.440	0.118
	MetS (no MetS vs. MetS)	2.831	16.967	5.013	57.427	20.715	<0.001
	Age at menopause	0.202	1.223	1.061	1.410	7.740	0.005
	Time since menopause (years)	0.231	1.260	1.110	1.431	12.814	<0.001
	BKMI (score)	0.044	1.045	0.958	1.139	0.970	0.325
Model 5	Intercept					65.565	<0.001
	MetS (no MetS vs. MetS)	2.626	13.812	4.972	38.373	25.365	<0.001

OR—odds ratio, CI—confidence interval, b—nonstandardized regression coefficient, BKMI—Blatt–Kupperman Menopausal Index, MetS—metabolic syndrome. Model 0—model adjusted for the variable: age at menopause. Model 1—model adjusted for the variables: age at menopause, time since menopause [years], Model 2—model adjusted for the variables: age at menopause, time since menopause [years], BKMI score Model 3—model adjusted for the variables: MetS, age at menopause, time since menopause [years], BKMI score. Model 4—model adjusted for the variables: abdominal obesity, MetS, age at menopause, time since menopause [years], BKMI score. Model 5—model adjusted for the variable: MetS.

## Data Availability

Data are contained within the article.
